# Advances in Radiation Oncology for Pancreatic Cancer: An Updated Review

**DOI:** 10.3390/cancers14235725

**Published:** 2022-11-22

**Authors:** Jason Liu, Percy Lee, Heather M. McGee, Vincent Chung, Laleh Melstrom, Gagandeep Singh, Mustafa Raoof, Arya Amini, Yi-Jen Chen, Terence M. Williams

**Affiliations:** 1 Department of Radiation Oncology, City of Hope National Medical Center, Duarte, CA 91010, USA; 2 Department of Radiation Oncology, City of Hope Orange County, Irvine, CA 92618, USA; 3 Department of Medical Oncology, City of Hope National Medical Center, Duarte, CA 91010, USA; 4 Division of Surgical Oncology, City of Hope National Medical Center, Duarte, CA 91010, USA

**Keywords:** radiotherapy, pancreatic cancer, locally advanced, borderline resectable, resectable

## Abstract

**Simple Summary:**

Pancreatic cancers are highly aggressive tumors that carry a poor prognosis. With recent advances in radiation therapy techniques and systemic therapy, there is hope that the treatment landscape for pancreatic cancers will improve in the near future. This review summarizes radiation dose escalation strategies to improve outcomes in locally advanced pancreatic cancer as well as novel neoadjuvant therapy strategies to improve outcomes in resectable and borderline resectable pancreatic cancers.

**Abstract:**

This review aims to summarize the recent advances in radiation oncology for pancreatic cancer. A systematic search of the MEDLINE/PubMed database and Clinicaltrials.gov was performed, focusing on studies published within the last 10 years. Our search queried “locally advanced pancreatic cancer [AND] stereotactic body radiation therapy (SBRT) [OR] hypofractionation [OR] magnetic resonance guidance radiation therapy (MRgRT) [OR] proton” and “borderline resectable pancreatic cancer [AND] neoadjuvant radiation” and was limited only to prospective and retrospective studies and metanalyses. For locally advanced pancreatic cancers (LAPC), retrospective evidence supports the notion of radiation dose escalation to improve overall survival (OS). Novel methods for increasing the dose to high risk areas while avoiding dose to organs at risk (OARs) include SBRT or ablative hypofractionation using a simultaneous integrated boost (SIB) technique, MRgRT, or charged particle therapy. The use of molecularly targeted agents with radiation to improve radiosensitization has also shown promise in several prospective studies. For resectable and borderline resectable pancreatic cancers (RPC and BRPC), several randomized trials are currently underway to study whether current neoadjuvant regimens using radiation may be improved with the use of the multi-drug regimen FOLFIRINOX or immune checkpoint inhibitors.

## 1. Introduction

Pancreatic cancer accounts for only 3% of cancer cases but is the 3rd leading cause of cancer mortality in the United States [1]. Notable improvements have been made over the past decade, particularly in regard to systemic therapy and radiation delivery technique. Generally, the treatment approach for pancreatic cancer depends on resectability status, with the National Comprehensive Cancer Network (NCCN) guidelines [2] defining three categories based on extent of invasion into the vasculature: resectable (RPC), borderline resectable (BRPC), and locally advanced (LAPC) disease. An effective multidisciplinary approach is the optimal strategy for achieving the best outcomes in patients with pancreatic cancer.

The purpose of this narrative review is to summarize recent advances in radiation oncology in pancreatic cancer. Contrary to other recently published reviews [3,4,5] on this topic (which have focused more on radiotherapy timing and modality selection, genomic testing and targeted therapy selection, and novel preclinical model development, respectively), our focus is on advancements in radiotherapy techniques allowing for dose escalation in LAPC, advancements in novel radiosensitizers to pair with radiation in LAPC, and advancements in neoadjuvant strategies using radiation for RPC and BRPC. While many of these advances are still investigational and have yet to be widely adopted, it is important to be aware of them as they may soon change the treatment landscape for pancreatic cancer.

## 2. Materials and Methods

A systematic search of the MEDLINE/PubMed database (accessed on 1 July 2022) was performed, focusing on studies published within the last 10 years. Our search queried “locally advanced pancreatic cancer [AND] stereotactic body radiation therapy (SBRT) [OR] hypofractionation [OR] magnetic resonance guidance radiation therapy (MRgRT) [OR] proton” as well as “resectable pancreatic cancer [AND] neoadjuvant radiation” and was limited only to prospective and retrospective studies and metanalyses, omitting abstracts, books, documents, and reviews. Our search resulted in 349 total references. These were manually reviewed, and only 43 references were within our scope of interest ranging from 2014–2022. Prospective randomized studies were prioritized as having the highest level of evidence, followed by prospective single-arm studies, followed by meta-analyses of retrospective studies, followed by retrospective studies.

## 3. Results

### 3.1. Locally Advanced Pancreatic Cancer

#### 3.1.1. Advances in Radiation Therapy Technique to Allow for Dose Escalation

A standard chemoradiation regimen using conventional fractionation for LAPC has been 54 Gy in 30 fractions. This dose fractionation was established by the LAP07 trial [6], which compared induction chemotherapy to chemoradiation after induction chemotherapy (capecitabine), showing a local control (LC) benefit with the addition of chemoradiation, but no overall survival (OS) benefit compared to induction chemotherapy alone. After a median follow up of 3 years, the LC rate in the chemoradiation arm was 68% (compared to 54% in the chemotherapy alone arm, *p* = 0.04), and the median OS was 15.2 months (compared to 16.5 months in the chemotherapy alone arm, *p* = 0.83). The recent presentation of the CONKO-007 trial [7] interim results using a dose of 50.4 Gy in 28 fractions similarly found no OS benefit with the addition of chemoradiation after induction chemotherapy, although there was a higher rate of pathologic complete response (pCR) and negative circumferential margin resection with the addition of chemoradiation compared to induction chemotherapy alone.

The reason why the LC benefit gained from chemoradiation did not translate into an OS benefit in the LAP07 trial is likely multifactorial, but it is believed in part that the improvements in LC were not substantial enough to impact OS [8]. It is hypothesized that dose escalation may further improve LC and ultimately improve OS in patients with LAPC, although this has yet to be demonstrated in several prospective studies. There is retrospective evidence to support the notion of dose escalation based on a study by Krishnan et al. [9], which found that escalating the chemoradiation dose to a biologically effective dose (BED) > 70 Gy resulted in improved OS (median 17.8 vs. 15.0 months, *p* = 0.03) and freedom from local progression (FFLP) (10.2 vs. 6.2 months, *p* = 0.05) compared to the standard chemoradiation dose of 54 Gy in 30 fractions. The median OS was 17.8 months in the high dose chemoradiation arm vs. 15.0 months in the standard dose chemoradiation arm (*p* = 0.03) and no additional toxicity was observed in the high dose group compared to the standard dose group.

Recent advances in radiation therapy technique have allowed for dose escalation in the form of SBRT. The standard SBRT dose used today for LAPC is 25–33 Gy in 5 fractions [2], but evidence suggests that dose escalation beyond 25–33 Gy is needed in order to achieve better outcomes compared to standard dose chemoradiation. A prospective phase II study by Herman et al. [10] using SBRT to a dose of 33 Gy in 5 fractions found similar outcomes to those seen when using standard dose chemoradiation. The 1-year FFLP was 78%, and the median OS was 13.9 months. Another retrospective study by Park et al. [11] also found similar outcomes when using SBRT to a dose of 33 Gy in 5 fractions compared to standard dose chemoradiation. The 1-year OS was 56.2% for SBRT and 59.6% for chemoradiation (*p* = 0.75). While SBRT in the 25–33 Gy range may still be a reasonable option, studies in other diseases, such as lung cancer, have shown that a total dose of 50 Gy or more in 5 fractions (or biological effective dose (BED) of >100 Gy with alpha/beta of 10) is needed for effective tumor control and is more akin to an ablative dose [12].

Dose escalation beyond 33 Gy in 5 fractions poses a challenge due to nearby organs at risk (OARs), including stomach and bowel. These viscous hollow structures are particularly sensitive to higher radiation doses. One method to dose escalate while respecting OARs and normal tissue constraints is to increase the number of fractions and use an ablative hypofractionation technique. Indeed, ultra-hypofractionation of the radiation dose has been proved highly effective and safe in other critical challenging cancer scenarios [13], being feasible also among older patients [14]. A retrospective study by Reyngold et al. [15] established the safety and efficacy of an ablative hypofractionation technique (BED ~98 Gy) in 119 patients with LAPC. Patients with tumors >1 cm from stomach or intestines received 67.5 Gy in 15 fractions, and patients with tumors <1 cm from stomach or intestines received 75 Gy in 25 fractions with concurrent fluoropyrimidine. All patients were treated using intensity-modulated radiation therapy (IMRT) technique. Elective nodal coverage included peripancreatic nodes within 1 cm of tumor, celiac, and superior mesenteric artery (SMA) nodes. Planning treatment volumes (PTVs) specified exclusion of OARs from high dose treatment volumes, which resulted in incomplete coverage of the gross tumor volume (GTV) by the ablative prescription dose along any direct tumor-OAR interface. Using this technique, the median OS was 18.4 months, and the 1-year FFLP was 82.4%, both of which were improved compared to historic controls of standard dose chemoradiation. The rate of grade 3 toxicities was 13%, with 8% of patients experiencing upper gastrointestinal (GI) bleeding, 2% of patients experiencing gastric outlet obstruction, and 3% of patients experiencing bile duct stenosis. A secondary analysis found improved toxicity using this technique compared to standard doses of 3D conformal radiation therapy (3D-CRT) [16]. Larger prospective studies are needed before this technique is performed outside of experienced centers.

An alternative way to dose escalate is to use a simultaneous integrated boost (SIB) approach. The advantage of an SIB approach is that the total tumor volume may receive one dose, while a particular high risk area far away from OARs may receive a higher dose in the same number of fractions. The tumor-vessel interface is a particularly high risk area that has been recommended to receive a SIB [17,18]. Koay et al. [19] described a method of using an SIB approach for both an ablative hypofractionation and SBRT. For both techniques, computed tomography (CT) simulation with intravenous (IV) contrast is recommended to be performed using end-expiratory breath hold. For SBRT, gold fiducial markers are recommended to be placed prior to CT simulation to help with daily image guidance. Regarding the dose and volumes for ablative hypofractionation, it is recommended that a 1 cm uniform expansion from the GTV, celiac artery, SMA, and superior mesenteric vein (SMV) should receive a dose of 37.5 Gy in 15 fractions while the GTV between the celiac artery and SMA receives a dose of 67.5 Gy. Regarding the dose and volumes for SBRT, it is recommended that a 3 mm uniform expansion from the GTV and tumor-vessel interface receives a dose of 33 Gy in 5 fractions while the GTV between the celiac artery and SMA receives a dose of 50 Gy. During plan evaluation, protection of surrounding OARs should take precedence over maximizing coverage for the high dose volume. Outcomes have not yet been reported using this technique.

Yet another way to dose escalate is to include an SBRT boost after chemoradiation. There is currently one prospective study by Parisi et al. [20] that included 13 patients with LAPC who were planned to be receive induction chemotherapy followed by standard dose chemoradiation followed by SBRT boost. Ultimately, only 8/13 patients were treated with this approach, with 3 patients having early hepatic progression disease, 1 patient having cardiovascular complications, and 1 patient having surgical radicalization. Among the 8 patients treated with this approach, the median SBRT dose was 12 Gy (range 10–21 Gy) in 1–3 fractions. None of the patients developed grade 3 acute or late toxicities. The median OS was 21.5 months, with a 2-year LC rate of 73%, both of which were improved compared to historic controls of standard dose chemoradiation alone. While the results of this study are promising, further prospective studies are needed to validate this approach.

Another method to dose escalate while respecting nearby OARs is to improve image guidance, particularly in the form of magnetic resonance guided radiation therapy (MRgRT). MRgRT affords superior soft tissue delineation, which is particularly important when the bowel/stomach (or other OARs) and tumor target are in close proximity and obviates the need for fiducial placement. Additionally, MRgRT platforms may allow advanced motion management and on-table, near real-time adaptive radiation capabilities. Two prospective and three retrospective studies have evaluated this method of dose escalation, with one study [21] showing a possible toxicity benefit and another study [22] showing a possible OS benefit with the use of MR guided dose escalation.

Regarding the three retrospective studies examining MRgRT, the first by Rudra et al. [21] treated 44 patients with LAPC with various types of MRgRT, i.e., SBRT alone or hypofractionated chemoradiation. Patients were stratified into high dose (BED > 70 Gy, *n* = 24) and standard dose (BED < 70 Gy, *n* = 20) groups. With a median follow-up of 17 months, patients in the high dose arm had significantly improved 2-year OS (49% vs. 30%, *p* = 0.03), but no difference in 2-year FFLP (77% vs. 57%, *p* = 0.15) compared to patients in the standard dose arm. There were no grade 3+ toxicities in the high dose arm, while there were three toxicities in the standard dose arm, leading to a hypothesis that using on-table adaptive planning in the higher dose arm led to reduction in grade 3+ events. Another study by Hassanzadeh et al. [23] treated 44 patients with MRI-guided adaptive SBRT to a dose of 50 Gy in 5 fractions. Tumor abutted OARs in 79.5% of patients, and tumor invaded OARs in 11.1% of patients. Reoptimization was performed for 93% of all fractions. Median OS was 15.7 months, and 1-year LC was 84.3%, comparable to historic controls of SBRT to 25–33 Gy. The rate of late grade 2 GI toxicities (duodenal perforation, antral ulcer, and gastric bleed) was 6.8%, and the rate of late grade 3 GI toxicities (gastrointestinal ulcers) was 4.6%. A third study by Chuong et al. [24] treated 35 patients with MRI-guided adaptive SBRT to a dose of 50 Gy in 5 fractions. Elective nodal radiation was delivered to 20 (57.1%) patients to a dose of 50 Gy in 5 fractions, although any portion overlapping with planning organs at risk volumes (PRVs) received a lower dose of 35 Gy. No patient had fiducial markers placed, and all were treated with continuous intrafraction reoptimization. With a median follow-up of 10.3 months, the 1-year OS rate was 58.9%, and the 1-year LC rate was 87.8%, similar to historic controls of SBRT to 25–33 Gy. Acute grade 2 nausea and anorexia occurred in 8.6% of patients, and acute grade 3 diarrhea was reported in 2.9% of patients. Late grade 3 bile duct stenosis occurred in 2.9% of patients, and no grade 4–5 toxicities were observed.

Of the two prospective MRgRT studies, the first by Kim et al. [25] was a single arm phase I study using a TITE-CRM design to determine the maximum tolerated dose of ablative hypofractionated chemoradiation in 26 patients with BRPC and LAPC. The radiation dose was escalated from 40–45 Gy in 25 fractions up to 60–67.5 Gy in 15 fractions. There was only 1 dose limiting toxicity observed, with the maximum tolerated dose being 67.5 Gy in 15 fractions. After a median follow up of 40.6 months, the median OS was 14.5 months, similar to historic controls of standard dose chemoradiation. The 2-year local progression free survival (LPFS) and distant metastasis free survival (DMFS) were 85% and 57%, respectively. Authors concluded that ablative hypofractionated chemoradiation up to 67.5 Gy in 15 fractions is safe with promising LPFS and DMFS. The second prospective MRgRT study called SMART [26] was an international phase II study assessing the safety and efficacy of MRI-guided adaptive SBRT in 136 patients with LAPC or BRPC,. Interim analysis [22] presented at ASTRO 2022 showed promising results, with no patients experiencing grade 3 or higher gastrointestinal toxicity in the first 90 days after SMART treatment. The 1-year OS rate was 94%, the 1-year LC rate was 83%, and the distant progression-free survival rate was 51%, all of which were improved compared to historic controls of standard dose SBRT. There is a planned phase III trial called LAP-ABLATE [27] sponsored by ViewRay that aims to compare induction chemotherapy ± SMART 50 Gy in 5 fractions in 267 patients with LAPC.

One final method of dose escalation is to use charged particle therapy (e.g., protons, carbon ions), which has a steep dose falloff outside the target due to the Bragg peak and may better spare surrounding OARs (although there is still range uncertainty at the edge of the beam). There are currently three retrospective studies examining the safety and efficacy of proton beam therapy (PBT). The first study by Takatori et al. [28] was a retrospective review of a prospective database and found favorable OS (77% at 1 year) and FFLP (82% at 1 year) in 91 patients treated with PBT to a dose of 67.5 GyE in 25 fractions with concurrent gemcitabine. However, there was an unacceptably high rate of radiation-induced ulcers, occurring in 45/91 (49.4%) of patients treated with this technique. The two other retrospective studies [29,30] similarly found favorable OS and FFLP with PBT. The first by Hiroshima et al. [29] found a median OS of 25.6 months and a 1-year LC rate of 83% in 42 patients using a dose of 54–67.5 GyE in 25–33 fractions. The other by Kim et al. [30] found a median OS of 26.1 months and a 1-year LC rate of 79% in 44 patients using a dose of 45–50 Gy in 10 fractions. Both studies showed minimal to no late grade 3+ GI toxicities using this technique. Regarding carbon ion therapy, there is one retrospective study by Kawashiro et al. [31] that treated 72 patients with LAPC to a dose of 52.8 Gy in 12 fractions. After a median follow up of 13.6 months, the median OS was 21.5 months, and the 1-year LC rate was 84%. The primary grade 3+ toxicity was hematologic (26% of patients), and only 1 patient developed late grade 3 GI toxicity (ascites). Further prospective studies are needed to better assess the safety and efficacy of proton therapy and carbon ion therapy. A summary of studies discussing recent advances in radiation therapy for LAPC can be seen in Table 1.

#### 3.1.2. Advances in Systemic Therapy for Improved Radiosensitization

Despite recent advancements in radiation therapy techniques, many patients with LAPC are not candidates for dose escalation due to tumor size, location, invasion into adjacent bowel, inability to control internal motion, and insufficient access to on-board imaging or adaptive radiation technology. Radiosensitizing agents offer a novel way to preferentially potentiate response to radiation in tumor cells while having less effect on normal tissues, thereby widening the therapeutic window. Several molecular agents have shown promise in pairing with radiation in preclinical and clinical studies, including epithelial growth factor (EGFR) inhibitors, cell cycle checkpoint inhibitors (Wee1, Chk1/2), DNA-dependent protein kinase (DNA-PK) inhibitors, ataxia telangiectasia mutated and Rad3-related (ATR) inhibitors, receptor tyrosine kinase (RTK) inhibitors, and KRAS pathway effector (MEK, ERK, PI3K, AKT, mTOR) inhibitors, among several others [32]. A low cost, animal free model has recently been developed, enabling the possibility of long-term in vitro hypoxic 3D cell culture for pancreatic cancer [33]. This novel platform for radiation treatment screening can be used for long-term in vitro post-treatment observations as well as for fractionated radiotherapy treatment.

Several clinical studies have examined the safety and efficacy of EGFR inhibitors paired with chemoradiation for LAPC. Results have generally shown that this combination therapy is safe and well tolerated, although it is unclear whether combination therapy improves outcomes. A prospective phase I dose escalation study by Jiang et al. [34] sought to define the dose-limiting toxicity and maximum tolerated dose of erlotinib concurrent with standard dose chemoradiation in 15 patients with LAPC. A total of four dose levels were designed, and it was found that treatment was well tolerated at the highest dose level of capecitabine 925 mg/m^2^ twice daily and erlotinib 100 mg daily. The median OS was favorable at 13.2 months, and the most frequent side effects were lymphopenia, nausea, vomiting, diarrhea, electrolyte imbalances, and skin rash, most of which were grade 1–2. A prospective phase II study by Crane et al. [35] examined the safety and efficacy of induction cetuximab, gemcitabine, and oxaliplatin followed by concurrent standard dose chemoradiation with cetuximab in 69 patients with LAPC. With a median follow-up time of 20.9 months, the median OS was 19.2 months, and 1-year FFLP was 77.2%, slightly better than historic controls of standard dose chemoradiation alone. The most common toxicities were GI (32% grade 2, 10% grade 3), fatigue (26% grade 2, 6% grade 3), and acneiform rash (54% grade 2, 3% grade 3). The recent PARC trial [36] was the first randomized study to show no improvement in outcomes with the addition of cetuximab to maintenance therapy. A total of 68 patients with LAPC were randomized to receive standard dose radiation (54 Gy in 30 fractions) with gemcitabine and cetuximab administered weekly, followed by either maintenance gemcitabine plus cetuximab vs. gemcitabine alone. Compared to historic controls, the addition of cetuximab to gemcitabine-based chemoradiation and maintenance chemotherapy did not improve OS or LC. The median OS was 13.1 months, and the 1-year LC rate was 76.6%. Grade 3+ nausea and GI hemorrhage were the most common non-hematologic toxicities seen in 13% and 7% of patients respectively. Further studies are needed to better potentiate the radiosensitizing effect of EGFR inhibitors when combined with chemoradiation.

The Wee1 checkpoint inhibitor adavosertib (AZD1775) has shown promise in improving outcomes in patients with LAPC when combined with chemoradiation. A prospective phase I dose escalation study by Cuneo et al. [37] sought to define the dose-limiting toxicity and maximum tolerated dose of adavosertib concurrent with standard dose chemoradiation in 34 patients with LAPC. Four dose levels were included, and it was found that the optimal dose was at the second highest dose level of 150 mg/day. Eight (24%) patients experienced a dose-limiting toxicity, half of which occurred at the highest dose level of 175 mg/day, with the most common dose-limiting toxicities being anorexia, nausea, and fatigue. The median OS was 21.7 months, and the 1-year FFLP was 84% in patients who received a dose level of 150 mg/day or above, which was numerically higher than historic controls of standard dose chemoradiation alone. Further Phase II studies are warranted with this approach. 

Encouraging results have also been shown using the poly(ADP-ribose) polymerase-1/2 (PARP) inhibitor veliparib in combination with chemoradiation, especially in a particular subset of patients with alterations in the expression of DNA damage repair proteins. A prospective phase I dose escalation study by Tuli et al. [38] sought to define the dose-limiting toxicity and maximum tolerated dose of veliparib concurrent with chemoradiation to a dose of 36 Gy in 15 fractions in 30 patients with LAPC. Six dose levels were included, and it was found that the second highest dose level of veliparib 40 mg twice daily and gemcitabine 400 mg/m^2^ was best tolerated. Dose limiting toxicities occurred in 12 (40%) of patients, with 7 occurring at the highest dose level, the most common being grade 3+ lymphopenia and anemia. The rate of grade 3+ toxicities (nausea, vomiting, diarrhea, abdominal pain, and colitis) was 34%. Median OS was 15 months for all patients and 19 months for patients with alterations in DNA damage repair proteins PARP3 and RBX1. Integration of genomic, transcriptomic, and protein based-biomarkers of response is likely to be important in maximizing the effect of this PARP inhibitor. Validation in larger prospective phase II trials is warranted.

Molecularly targeted agents have also been tested in combination with SBRT to improve radiosensitization. A prospective phase I dose escalation study by Lin et al. [39] sought to define the dose-limiting toxicity and maximum tolerated dose of nelfinavir (HIV protease inhibitor and AKT inhibitor) concurrent with SBRT to a dose of 25–40 Gy in 5 fractions in 46 patients with BRPC and LAPC. A total of six dose levels were designed, and it was found that treatment was well tolerated at the highest dose level of nelfinavir 1250 mg twice daily with SBRT to 40 Gy in 5 fractions. The rate of grade 3+ GI bleeding was 10.8%. The median OS was 14.4 months, and the LC rate was 85% at 1 year. A total of 13 patients were considered resectable after chemoradiation, of which 9 had initially BRPC and 4 had initially LAPC. All but 1 patient underwent surgery, and 11/12 (91.7%) achieved negative margins. This treatment regimen appears to be safe and effective, although careful attention to treatment planning parameters is recommended to reduce the incidence of GI bleeding. Another molecularly targeted agent currently being studied in combination with SBRT is the small molecule dismutase mimetic avasopasem (GC4419), which is supposed to function as a radioprotector to protect normal tissues surrounding the irradiated area. A randomized trial [40] of 47 patients with LAPC treated with SBRT in combination with avasopasem vs. placebo was recently completed January 2022, although results have yet to be published.

The role of immunotherapy in combination with radiation therapy is also an active area of investigation in LAPC. Currently, no studies exist showing a benefit with immunotherapy in LAPC, although a recent phase II randomized trial by Zhu et al. [41] in locally recurrent pancreatic cancer after resection showed an OS benefit in patients treated with SBRT, pembrolizumab and trametinib as compared to SBRT plus gemcitabine. The median OS was 14.9 months vs. 12.8 months (*p* = 0.02). Toxicity appeared to be similar between treatment arms, with grade 3+ hepatotoxicity occurring in 12% vs. 7%, grade 3+ neutropenia occurring in 1% vs. 11%, and serious adverse events occurring in 22% vs. 14% of patients, respectively. Results of ongoing clinical trials [42,43,44] examining the safety and efficacy of combination immunotherapy with radiation in LAPC are eagerly awaited. A summary of studies discussing recent advances in systemic therapy for radiosensitization for LAPC can be seen in Table 2.

### 3.2. Resectable and Borderline Resectable Pancreatic Cancer

#### Advances in Neoadjuvant Strategies Using Radiation

Upfront surgery followed by adjuvant chemotherapy has long been the standard of care in patients with resectable pancreatic cancer. Unfortunately, less than half of patients with localized pancreatic cancer will receive the intended therapy, with up to 20% having metastatic or unresectable disease at the time of surgery [45] and up to 50% will not be able to recover from surgery sufficiently or in time to tolerate adjuvant chemotherapy, leading to local or distant recurrence [46]. Neoadjuvant therapy has been proposed as a way to allow more patients to receive systemic therapy and avoid futile surgeries. Two recent phase II/III randomized trials have suggested a benefit with neoadjuvant chemoradiation over upfront surgery. The first was a Korean study by Jang et al. [47] that closed early after finding an OS benefit in patients with BRPC who received neoadjuvant vs. adjuvant chemoradiation (21 months vs. 12 months, *p* = 0.03). The resection rate was 71% vs. 78%, and the R0 resection rate was 52% vs. 26% in the neoadjuvant chemoradiation vs. upfront surgery arm. The second was the PREOPANC-1 trial [48], which found an improvement in R0 resection rate (71% vs. 40%), disease free survival (DFS), and FFLP with the use of neoadjuvant gemcitabine followed by chemoradiation (36 Gy in 15 fractions) before surgery, but not OS (16 months vs. 14.3 months, *p* = 0.10). A subset analysis did show an OS benefit in patients with pre-specified BRPC (17.6 months vs. 13.2 months, *p* = 0.03) but not in patients with pre-specified RPC (14.6 months vs. 15.6 months, *p* = 0.83). A French retrospective study [49] and a metanalysis by Cloyd et al. [50] similarly found improvements in OS with the use of neoadjuvant therapy vs. upfront surgery in patients with RPC and BRPC. Building upon these successful neoadjuvant regimens and enhancing the effect of radiation are ongoing areas of investigation.

One area of active study is whether the multi-drug regimen FOLFIRINOX may improve outcomes and better sensitize tumors to radiation in the neoadjuvant setting. In metastatic pancreatic cancer, FOLFIRINOX has been shown to improve OS compared to gemcitabine based on a landmark study by Conroy et al. [51], but very few studies have tested its use in the neoadjuvant setting. Alliance A021501 [52] is one study that prospectively examined the safety and efficacy of neoadjuvant mFOLFIRINOX with or without subsequent radiation in 126 patients with BRPC. Patients were randomized to receive either neoadjuvant mFOLFIRINOX × 8 cycles (arm A) or neoadjuvant mFOLFIRINOX × 7 cycles followed by SBRT to a dose of 33–40 Gy in 5 fractions or 25 Gy in 5 fractions (arm B). The 18-month OS rate for arm A was 66.7%, improved compared to historic data using a cutoff of >63%, while the 18-month OS rate for arm B was 47.3%, not improved compared to historic data. The rate of grade 3+ toxicity was 57% in arm A vs. 64% in arm B. It is unclear why patients who received radiation had worse outcomes compared to patients who received mFOLFIRINOX alone, although there are several concerns with the study. One concern is that so few patients in the SBRT arm eventually underwent surgery (51%) compared to historic controls (68% in Alliance A021101 [53], 71% in Jang et al. [47], and 61% in PREOPANC-1 [48]). In addition, the trial design used R0 rate as the stopping point and this caused arm B to close early. There were also imbalances in the treatment of arm A and B, with arm B having a higher rate of mFOLFIRINOX treatment delays (49% vs. 60%) and dose reductions (60% vs. 75%) compared to arm A. The aforementioned French retrospective study [49] had actually found opposite results, with the addition of radiation after FOLFIRINOX being associated with better OS and other pathological outcomes. Further study is needed to better clarify the interaction between FOLFIRINOX and radiation in the neoadjuvant setting.

To assess whether neoadjuvant FOLFIRINOX may be better than the current standard of care using chemoradiation, two highly anticipated randomized clinical trials are currently underway. The first, known as PREOPANC-2 [54], is a phase III trial that randomizes patients with RPC and BRPC to receive neoadjuvant FOLFIRINOX × 8 cycles followed by surgery without adjuvant treatment (arm A) vs. neoadjuvant gemcitabine x 3 cycles followed by hypofractionated chemoradiation to a dose of 36 Gy in 15 fractions (PREOPANC-1-like regimen) followed by surgery and adjuvant gemcitabine x 4 cycles (arm B). The primary endpoint is OS, and the secondary endpoints include DFS, quality of life, resection rate, and R0 resection rate. The second known as ESPAC-5F [55] is a four-arm phase II trial that randomizes patients with BRPC to receive immediate surgery (arm 1) or neoadjuvant therapy of either gemcitabine and capecitabine × 2 cycles (arm 2), FOLFIRINOX × 4 cycles (arm 3), or capecitabine-based chemoradiation to a dose of 50.4 Gy in 28 fractions (arm 4). The primary endpoint is the resection rate. Secondary endpoints include OS and toxicity. An interim analysis was recently published in abstract form, which found an improvement in the 1-year OS rate with neoadjuvant therapy vs. immediate surgery (77% vs. 40%, *p* < 0.01), but no difference in resection rate (62% vs. 55%, *p* = 0.67) or R0 resection rate (15% vs. 23%, *p* = 0.72) between the two groups [55]. The interim analysis did not include results comparing outcomes between the various neoadjuvant therapy arms. The results of both of these ongoing clinical trials are eagerly awaited. 

Another area of active study is whether the addition of immune checkpoint inhibitors to neoadjuvant chemoradiation may improve outcomes. A phase II study by Rahma et al. [56] randomized 37 patients with RPC and BRPC to receive pembrolizumab concurrently with chemoradiation (50.4 Gy in 28 fractions plus capecitabine) vs. chemoradiation alone prior to resection. The primary endpoints were safety and differences in tumor-infiltrating lymphocyte (TIL) density assessed using multiplexed immunofluorescence on resected tumor specimens. After neoadjuvant therapy, 9/24 (37.5%) patients in the experimental arm had unresectable disease compared to 4/13 (30.8%) patients in the control arm. A total of 24 patients eventually underwent surgery and were evaluable for the primary endpoint. The mean difference in TIL density between the two treatment arms was 36 cells/mm2 (95% confidence interval −85–157, *p* = 0.48). Additional analysis did not show any difference in the density of activated cytotoxic T cells, regulatory T cells, macrophages, or granulocytes. With a median follow-up time of 26.4 months, the median OS was 27.8 months in the experimental arm vs. 24.3 months in the control arm (*p* = 0.68). The most common grade 3+ toxicities were lymphopenia (29% vs. 31%) and diarrhea (8% vs. 0%). While preliminary results did not show any improvement in clinical outcomes or TIL density with the addition of pembrolizumab to neoadjuvant chemoradiation, more evidence is needed before any conclusions are drawn. Larger prospective phase II trials [57] are currently underway to better understand the benefit of immunotherapy paired with chemotherapy and radiation in the neoadjuvant setting. In addition, radiation and PD-1 inhibitors have been combined with other immunomodulatory agents, including cancer vaccines such as GVAX, which is a cancer vaccine composed of whole tumor cells genetically modified to secrete granulocyte-monocyte colony-stimulating factor (GM-CSF). A phase II study [58] is currently underway testing this treatment combination. A summary of studies discussing recent advances in neoadjuvant therapies for RPC and BRPC can be seen in Table 3.

## 4. Limitations

While this review provides a semi-comprehensive overview of recent advances in radiation oncology for pancreatic cancer, there are several limitations. First, there is the inability to discern a true treatment effect if there are differing results based on various studies. We have attempted to explain why some studies may show a positive result while others show a negative result based on differing eligibility criteria or treatment details; however, this is ultimately prone to bias, as we authors may inherently favor a positive treatment effect over a negative treatment effect. Secondly, the level of evidence available for analysis is not the strongest, with only a limited number of phase I/II studies published in the literature. Should larger phase III trials be published in the future, the results of those findings should supersede any conclusions drawn from a review of phase I/II studies. Lastly, there is the possibility of selection bias, information bias, and confounding bias common in all reviews. 

## 5. Conclusions

Pancreatic cancer is a highly aggressive disease that has a historically dismal prognosis. Notable improvements have been made over the past decade, particularly in regard to radiation therapy technique and systemic therapy. For LAPC, the current recommended treatment is still standard dose chemoradiation to 54 Gy in 30 fractions or SBRT to 25–33 Gy in 5 fractions. However, anticipated changes within the next several years may include dose escalation in the form of SBRT to 50 Gy in 5 fractions or ablative hypofractionation to 67.5 Gy in 15 fractions using an SIB technique, MRgRT, or charged particle therapy (such as proton therapy or carbon ion therapy). These techniques should only be used at experienced centers currently. The use of molecularly targeted agents with radiation to improve radiosensitization and widen the therapeutic window has also shown promise in several prospective phase I/II studies, but larger phase III studies are needed before they become implemented in everyday practice. For BRPC, the current recommended treatment is neoadjuvant chemotherapy ± radiation followed by surgery. Several randomized trials are currently underway to study whether current neoadjuvant regimens may be improved with the use of the multi-drug regimen FOLFIRINOX or immune checkpoint inhibitors with or without radiation. The optimal neoadjuvant regimen may possibly be identified within the next several years. Additional work is needed to further improve optimal radiation and chemoradiation strategies to improve outcomes for patients with pancreatic cancer.

## Figures and Tables

**Table 1:** Advances in radiation technique to allow for dose escalation in LAPC.

Study	Design	Patients	Intervention	OS	FFLP	Toxicity
LAP07 [6]	Phase III	133	Standard dose chemoradiation (54 Gy in 30 fractions plus capecitabine)	Median 15.2 months	68%	6% grade 3+ nausea
Herman et al. [10]	Phase II	49	Standard dose SBRT (33 Gy in 5 fractions)	Median 13.9 months	78% at 1 year	2% grade 2+ gastritis and ulcers
Krishnan et al. [9]	Retrospective	47	Dose escalated chemoradiation (BED > 70 Gy plus gemcitabine or capecitabine)	Median 17.8 months	Median 10.2 months	2% grade 3+ nausea
Reyngold et al. [15]	Retrospective	119	Ablative hypofractionation (67.5 Gy in 15 fractions or 75 Gy in 25 fractions plus fluoropyrimidine)	Median 18.4 months	83% at 1 year	13% grade 3+ toxicity (8% GI bleeding, 2% gastric outlet obstruction, 3% bile duct stenosis)
Parisi et al. [20]	Phase I/II	8	Induction chemotherapy followed by standard dose chemoradiation followed by SBRT boost to a median dose of 12 Gy in 1–3 fractions	Median 21.5 months	73% at 2 years	No grade 3 toxicities
Rudra et al. [21]	Retrospective	20	High dose MRgRT (BED > 70 Gy, SBRT alone or hypofractionated RT plus concurrent gemcitabine, capecitabine, or gemcitabine-nab-paclitaxel)	Median 20.8 months	77% at 2 years	0% grade 3+ GI toxicities
Hassanzadeh et al. [23]	Retrospective	44	MR guided SBRT (50 Gy in 5 fractions) with adaptive reoptimization	Median 15.7 months	84% at 1 year	5% grade 3+ ulcers
Chuong et al. [24]	Retrospective	35	MR guided SBRT (50 Gy in 5 fractions) with adaptive reoptimization	59% at 1 year	88% at 1 year	6% grade 3+ diarrhea and bile duct stenosis
TITE-CRM (Kim et al.) [25]	Phase I/II	26	MR guided radiation therapy (40–45 Gy in 25 fractions up to 60–67.5 Gy in 15 fractions) with full-dose gemcitabine/nab-paclitaxel	Median 14.5 months	85% at 2 years	14% dose limiting toxicity (cholecystitis)
SMART (Parikh et al.) [22]	Phase II	136	MR guided SBRT (50 Gy in 5 fractions) with adaptive reoptimization	94% at 1 year	83% at 1 year	No grade 3 toxicities
Takatori et al. [28]	Retrospective review of prospective database	91	Proton beam therapy (67.5 GyE in 25 fractions plus gemcitabine)	77% at 1 year	82% at 1 year	50% grade 3+ ulcers
Hiroshima et al. [29]	Retrospective	42	Proton beam therapy (54–67.5 GyE in 25–33 fractions plus gemcitabine or S-1)	Median 25.6 months	83% at 1 year	0% grade 3+ GI toxicities
Kim et al. [30]	Retrospective	44	Proton beam therapy (45–50 GyE in 10 fractions plus gemcitabine, capecitabine, or gemcitabine-nab-paclitaxel)	Median 26.1 months	79% at 1 year	0% grade 3+ GI toxicities
Kawashiro et al. [31]	Retrospective	72	Carbon ion therapy (52.8 Gy in 12 fractions plus gemcitabine)	Median 21.5 months	84% at 1 year	0% grade 3+ GI toxicities

FFLP = freedom from local progression; OS = overall survival; SBRT = stereotactic body radiation therapy; MRgRT = MR-guided radiation therapy.

**Table 2:** Advances in systemic therapy for improved radiosensitization in LAPC.

Study	Phase	Patients	Intervention	OS	FFLP	Toxicity
LAP07 [6]	III	133	Standard dose chemoradiation (54 Gy in 30 fractions plus capecitabine)	Median 15.2 months	68%	6% grade 3+ nausea
Herman et al. [10]	II	49	Standard dose SBRT (33 Gy in 5 fractions)	Median 13.9 months	78% at 1 year	2% grade 2+ gastritis and ulcers
Jiang et al. [34]	II	15	Erlotinib (EGFR inhibitor) plus chemoradiation (50.4 Gy in 28 fractions plus capecitabine)	Median 13.2 months	Not reported	0% grade 3+ GI toxicities
Crane et al. [35]	II	69	Cetuximab (EGFR inhibitor) plus chemoradiation (50.4 Gy in 28 fractions plus capecitabine)	Median 19.2 months	77% at 1 year	10% grade 3+ GI toxicities
PARC [36]	II	34	Cetuximab (EGFR inhibitor) plus chemoradiation (54 Gy in 30 fractions plus gemcitabine)	Median 13.1 months	77% at 1 year	13% grade 3+ nausea and GI bleeding
Cuneo et al. [37]	II	34	Adavosertib (Wee1 inhibitor) plus chemoradiation (54 Gy in 30 fractions plus gemcitabine)	Median 21.7 months	84% at 1 year	24% grade 3+ anorexia, nausea, and fatigue
Tuli et al. [38]	II	30	Veliparib (PARP inhibitor) plus chemoradiation (36 Gy in 15 fractions plus gemcitabine)	Median 15 months for all, but 19 months in patients with PARP3 and RBX1 alterations	Not reported	34% grade 3+ nausea, vomiting, diarrhea, abdominal pain, colitis
Lin et al. [39]	II	46	Nelfinavir (AKT inhibitor) plus SBRT (40 Gy in 5 fractions)	Median 14.4 months	85% at 1 year	11% grade 3+ GI bleeding

FFLP = freedom from local progression; OS = overall survival; GI = gastrointestinal; EGFR = epidermal growth factor; PARP = poly (ADP-ribose) polymerase.

**Table 3:** Advances in neoadjuvant strategies using radiation in RPC and BRPC.

Study	Phase	Patients	Intervention	OS	Resection Rate	R0 Rate
PREOPANC-1 [48]	III	119, RPC and BRPC	Neoadjuvant gemcitabine × 3 cycles followed by chemoradiation (36 Gy in 15 fractions plus gemcitabine) followed by surgery followed by adjuvant gemcitabine × 6 cycles	Median 14.6 months for RPC and 17.6 months for BRPC	61%	71%
Alliance A021501 [52]	II	126, BRPC	Neoadjuvant mFOLFIRINOX × 8 cycles (arm A) vs. mFOLFIRINOX × 7 cycles followed by radiation (33–40 Gy in 5 fractions or 25 Gy in 5 fractions) (arm B) followed by surgery followed by adjuvant mFOLFOX × 6 cycles	Median 29.8 months for arm A vs. 17.1 months for arm B	58% for arm A 51% for arm B	88% for arm A 74% for arm B
PREOPANC-2 [54]	III	Goal of 368, RPC and BRPC	Neoadjuvant FOLFIRINOX × 8 cycles followed by surgery followed by no adjuvant treatment (arm A) vs. gemcitabine × 3 cycles followed by chemoradiation (36 Gy in 15 fractions plus gemcitabine) followed by surgery followed by adjuvant gemcitabine × 4 cycles (arm B)	Pending	Pending	Pending
ESPAC-5F [55]	II	88, BRPC	Immediate surgery (arm 1) vs. neoadjuvant gemcitabine and capecitabine × 2 cycles (arm 2) vs. neoadjuvant FOLFIRINOX × 4 cycles (arm 3) vs. chemoradiation (50.4 Gy in 28 fractions plus capecitabine) (arm 4) followed by surgery	40% at 1 year for arm 1 77% at 1 year for arms 2–4	62% for arm 1 55% for arms 2–4	15% for arm 1 23% for arms 2–4
Rahma et al. [56]	II	37, RPC and BRPC	Neoadjuvant pembrolizumab (arm A) plus chemoradiation (50.4 Gy in 28 fractions plus capecitabine) vs. chemoradiation alone (arm B) followed by surgery	Median 27.8 months in arm A and 24.3 months in arm B	64% for arm A 69% for arm B	Not reported

RPC = resectable pancreatic cancer; BRPC = borderline resectable pancreatic cancer; OS = overall survival.
